# Delivering quality care to all mothers and newborns requires governments to engage the private sector

**DOI:** 10.1136/bmj-2022-071650

**Published:** 2023-05-05

**Authors:** Rajat Chabba, Chantelle Allen, Nuhu Yaqub, Samantha R Lattof, Ramakrishnan Ganesan, Blerta Maliqi

**Affiliations:** 1 Market Solutions, Jhpiego, Baltimore, Maryland, USA; 2 Primary Health Care, Jhpiego, Baltimore, Maryland; 3 Department of Maternal, Newborn, Child, and Adolescent Health and Aging, World Health Organization, Geneva, Switzerland; 4 Maila Health, Berlin, Germany; 5 Abt Associates, Chennai, India

## Abstract

More structured engagement with the private sector will contribute to achieving universal health coverage with quality, argue **Rajat Chabba and colleagues**

In low and middle income countries the private healthcare sector is increasingly the first point of contact for women, men, and children seeking healthcare, irrespective of their socioeconomic status. In countries with mixed health systems,^1^ comprising both public and private sectors, the private sector is a key source of maternal and newborn health services, information, and products (box 1).^2^ ^3^ Cross sectional survey evidence from 57 low and middle income countries published in 2016 showed that the private sector accounted for a mean market share of 44% among users of antenatal care and a mean market share of 40% for care during childbirth.^2^ With an increasing proportion of mothers and newborns using private sector health services, researchers examining quality of care across the public and private sectors in low and middle income countries conclude that improvements in quality of care are warranted in both sectors.^4^

Box 1 **What is the private health sector?**

The private health sector includes non-governmental organisations and individuals that are directly and indirectly involved in the provision of health information, products, and services at the household, community, and facility levels, and are neither owned nor directly controlled by governments.^28^ This includes a wide range of for-profit and not-for-profit entities, formal and informal providers, faith based institutions, supply chain actors, and other intermediaries.

Many care seekers in low and middle income countries associate private sector health services with higher quality, but the quality of services provided in the private sector varies.^4^ ^5^ While country efforts and global strategies to improve maternal and newborn health acknowledge the role of the private sector in delivering quality services,^6^ ^7^ ^8^ the actions of low and middle income country governments to improve quality of care tend to focus on strengthening services within the public health sector alone as opposed to engaging with both sectors. Reasons for limited government engagement with the private sector—or forging partnerships between the public and private sectors to achieve specific goals^9^—vary from absence of policy frameworks and limited government expertise or experience, to lack of understanding of each sector’s intentions.^10^ However, achieving universal health coverage with quality requires working with everyone concerned in delivering care.^11^ ^12^ Some studies show success in delivering and improving quality of care in the private sector, particularly where regulatory and subsidy systems integrate the private sector in service delivery.^10^ The fact that a growing proportion of women seek and receive healthcare from private sector providers means that governments need to engage with and govern all health service providers—public and private, formal and informal—across the health system to deliver quality maternal and newborn healthcare and achieve universal health coverage.

Governments clearly have an opportunity to improve health outcomes by engaging the private sector in efforts to deliver quality care. We argue that to achieve universal health coverage with quality, governments must engage the private sector in a more structured way. Examples of this engagement include agreeing on common goals, developing and implementing policy, leveraging financing, and strengthening regulatory mechanisms to support service delivery.

## Private sector engagement can improve quality

When governments partner with the private sector to deliver care, it is usually with the goal of improving access, increasing health service utilisation, and enabling the continuity and expansion of services to settings where public health services are minimal or non-existent.^13^ Governments use different instruments to engage the private sector in service delivery. These vary from adopting financing mechanisms to creating public-private partnerships and service delivery models. For example, in Sudan the Federal Ministry of Health engaged regulated and licensed private providers to immunise children as part of the national immunisation strategy. Using government supplied vaccines, by 2017 the private sector had delivered 15.7% of all diphtheria, tetanus, pertussis vaccinations free in hard-to-reach and conflict affected areas in Sudan. This partnership model contributed to the achievement of 95% vaccine coverage in 2017 from a baseline of 62% in 2000. The experience of engaging the private sector in Sudan also showed the importance of incorporating the private providers into the national immunisation programme’s supervision and quality assurance system to achieve joint immunisation goals and ensure compliance with immunisation guidelines and policy.^13^

The role of the private sector in supporting continuity of essential services in low and middle income countries was also evident during the covid-19 pandemic. In 2021, data from 24 such countries surveyed by the Global Financing Facility, a global multi-stakeholder partnership in health and development, showed that the pandemic had disrupted the delivery of essential maternal and newborn health services, thus risking the reversal of hard earned gains in these services.^14^ In some countries, private providers helped to sustain essential services, ensuring surge capacity to deal with the increasing health system burden. In Mexico, for example, the Mexican Institute of Social Security contracted out private hospitals to provide childbirth services so that public hospital beds could be used for covid-19 patients.^15^ Service delivery data from Bangladesh documented that the private sector was able to maintain pre-pandemic population level service coverage of facility based delivery, antenatal care, and postnatal care services at the beginning of the pandemic.^16^

While these examples highlight the ways in which private sector engagement can supply or extend access to essential health services, quality of care provided in the private sector is highly variable. For example, a two phase baseline assessment conducted under the Manyata initiative from November 2016 to March 2017 and December 2017 to March 2018 in 201 private healthcare facilities across three states in India found that the overall quality of maternity care was poor, especially for clinical standards related to management of maternal and newborn complications.^5^ Between 2016 and 2019 the Manyata initiative engaged 466 private facilities in quality improvement activities, and as a result the quality of clinical service delivery and adherence to multiple quality standards, such as management of eclampsia/pre-eclampsia and neonatal resuscitation, substantially increased from 29% to 93% during the intervention period.^17^ The varying quality among private sector providers further justifies the need for governments to engage the private sector in more structured ways, such as by setting common goals, enacting regulatory mechanisms, policy making, and the implementation of quality of care standards.

## Governments must adopt a more structured approach to engaging with the private sector

A 2020 private sector engagement analysis by the World Health Organization reported that governments in low and middle income countries are struggling to engage structurally with the private health sector.^10^ For example, an analysis in the report of private sector engagement in 18 low and middle income countries across six World Health Organization regions with high coverage and utilisation of private health providers found that while governments recognised the private health sector’s role in achieving population health goals, specific policies on private sector engagement and formal dialogue mechanisms were uncommon. The analysis also found that while most of the 18 low and middle income countries had a public insurance system for healthcare, only half provided partial coverage for services provided by private health providers.^10^

This diversity of engagement also reflects, to some degree, the difference in maturity, growth, scale, and baseline quality in the private health sector across a range of low and middle income countries.^10^ For example, the motivation of governments to engage the private sector depends on the proportion of healthcare delivered by the private sector (large proportion of market share versus emerging private sector), the level of unmet need for maternal and newborn health services in the public sector, along with consumer demand for private healthcare.^10^ For its part, the motivation of the private sector to engage with the government is defined by a combination of these factors, along with an intrinsic motivation to contribute to social impact.^18^ However, even if motivated, the ability of governments to engage the private sector in delivering quality care depends on the existence or implementation of regulatory and financing mechanisms that facilitate collaboration and partnership, as well as expertise to engage the private sector.^10^ ^19^ Multi-stakeholder policy dialogues on private sector engagement to deliver quality of care for maternal and newborn health conducted in Ghana (2020) and Nigeria (2021) emphasised the need for more structured engagement with government through the development of financial mechanisms, such as tax rebates, loans, or guarantees to support private providers’ ability to deliver quality care.^20^ The Global Financing Facility has also invested in multiple financing tools and instruments such as blended financing, impact bonds, and capital market instruments to enable countries to engage the private providers in a more structured way in delivering quality maternal and newborn health services. For example, in Côte d’Ivoire, the Global Financing Facility supported private providers to form an umbrella association to enable their inclusion in performance based financing contracts with the government that include quality of care as a key outcome.^21^ ^22^

Other structured engagement mechanisms, supported by enabling policies, can facilitate increased access to and utilisation of quality health services. Examples vary from governments’ strategic purchasing and contracting out of primary health services to non-governmental organisations, such as the experience of the People’s Primary Healthcare initiative in Pakistan, to formal memorandums of understanding with private providers.^10^ In Ghana, the Ministry of Health has a memorandum of understanding with the Christian Health Association of Ghana, the country’s largest faith based healthcare provider, to deliver services in underserved communities.^23^ In addition to receiving technical support (such as new treatment guidelines and training) from the government, the health association appears in the Ministry of Health’s organogram and has participated in the development and implementation of national health strategies and plans. Providing 30%-40% of health services across Ghana, the health association also reports data in the routine information system and implements new government technical guidelines, including those on quality.^23^ However, other private for-profit providers in Ghana are yet to be engaged in the same way, which has led to a lack of incentives to report their data to the Ministry of Health and limited involvement in governance for quality. The successful experience of engaging the Christian Health Association of Ghana reflects the importance of structured engagement through regulatory mechanisms, such as memorandums of understanding and strategic public-private partnerships to achieve agreed health goals, including to strengthen the quality of maternal and newborn health services.

It is clear that governments in low and middle income countries need to focus on strengthening the public health system to achieve universal health coverage.^24^ While investing in the public health system to deliver quality care and achieve universal health coverage should be a priority for all governments, it is also important to recognise that in mixed health systems, healthcare is not delivered by the public sector alone.^10^ All providers across public and private sectors have a role in improving quality of maternal and newborn healthcare, and given the private sector also provides an increasing market share of maternal and newborn health services in many low and middle income countries, this further reinforces the need for a structured engagement of the private sector.^20^

## Actions to strengthen structured engagement with the private sector

To accelerate progress towards achieving universal health coverage, low and middle income countries need to explore new ways of governing mixed health systems by developing formal mechanisms and strategies to engage the private sector in a more structured way in the delivery of health services. As a first step, policy makers, the private sector, and implementers can draw on the approaches and tools provided in the World Health Organization’s 2020 strategy report on engaging the private health service delivery sector to strengthen the governance of mixed health systems and create enabling policy environments to deliver quality of care.^11^

With varying degrees of private sector presence, consumer demand, purchasing power, and country specific regulatory environment, private sector engagement strategies to deliver quality maternal and newborn health services need to be rooted in country context. Strategies that work in low and middle income countries with large and proved private sectors with mature regulatory environments, such as in India, will be different from those needed in countries with an emerging private sector and evolving regulatory environments, such as in Kenya and Ethiopia, or in fragile contexts, such as in countries with fragmented governance systems. More structured engagement with private sector entities may help governments tap into financial innovations that reflect the unique needs of their countries. mTIBA, for example, is a three way digital health platform that connects 4.7 million patients to over 5000 healthcare providers and payers (governments, insurers) in Kenya and could enable greater financial transparency in accessing health services across public and private sectors.^25^ ^26^ The scale, reach, and leadership of the public sector along with the flexibility, innovations, and resources of the private sector can accelerate the progress towards access to quality health services and universal health coverage in low and middle income countries.^27^

Critically, if quality of care is to improve across mixed health systems, further research is needed urgently to help identify the effectiveness of engagement strategies across the private sector within and also between countries.

The global goal for achieving universal health coverage is only a few years away. Country led meaningful engagement with the private sector is one more step towards creating an enabling environment to deliver quality universal health coverage across all health service providers. More structured engagement with the private sector, such as through establishing common health goals and building capacities and mechanisms for partnerships that reflect the needs of countries, is necessary if governments are to maximise the opportunities to deliver universal health coverage with quality.

**Key messages**

- The private sector includes important providers of maternal and newborn healthcare services in mixed health systems
- Efforts to improve quality of care need to engage all actors across both the public and private sectors
- Building a conducive policy environment supported by the right mechanisms and systems for private sector engagement will facilitate the achievement of universal health coverage with quality

## Notes

Contributors and sources: RC is medical doctor and management expert, working towards developing innovative healthcare models in low and middle income countries. RC is the senior technical adviser for Market Solutions at Jhpiego. CA’s work focuses on strengthening primary healthcare and she has practical experience working as a nurse in Asia and Africa. NY is a medical officer in in charge of paediatric quality of care at the World Health Organization. SRL has a special interest in improving maternal and newborn health by bridging research and technology. She has an MSc in global health and a PhD in population studies and demography. RG is experienced in leading programmes and assessments. BM manages the World Health Organization based secretariat of the Network for Improving Quality of Care for Maternal, Newborn, and Child Health. She is a medical doctor with a PhD in health systems management development and a masters in health management, planning, and policy. SRL wrote the first draft and reviewed the literature with inputs from BM and NY. All authors reviewed the first draft. BM and RC updated the outline, reviewed the literature, and wrote the second draft of the paper. NY and CA provided detailed inputs, additional literature, and editing to the second draft. All authors reviewed the second draft. BM finalised the draft with RC and all coauthors reviewed and agreed with the final version. BM is the guarantor of the paper.

Provenance and peer review: Commissioned; externally peer reviewed.

This article is part of a collection proposed by the World Health Organization and World Bank and commissioned by *The BMJ*. *The BMJ* peer reviewed, edited, and made the decision to publish these articles. Article handling fees are funded by the Bill and Melinda Gates Foundation. Jennifer Rasanathan, Juan Franco, and Emma Veitch edited this collection for *The BMJ*. Regina Kamoga was the patient editor.
